# Child Feces Disposal Practices in Rural Orissa: A Cross Sectional Study

**DOI:** 10.1371/journal.pone.0089551

**Published:** 2014-02-20

**Authors:** Fiona Majorin, Matthew C. Freeman, Sharmani Barnard, Parimita Routray, Sophie Boisson, Thomas Clasen

**Affiliations:** 1 Faculty of Infectious and Tropical Diseases, London School of Hygiene and Tropical Medicine, London, United Kingdom; 2 Department of Environmental Health, Rollins School of Public Health, Emory University, Atlanta, Georgia, United States of America; Stanford University, United States of America

## Abstract

**Background:**

An estimated 2.5 billion people worldwide lack access to improved sanitation facilities. While large-scale programs in some countries have increased latrine coverage, they sometimes fail to ensure optimal latrine use, including the safe disposal of child feces, a significant source of exposure to fecal pathogens. We undertook a cross-sectional study to explore fecal disposal practices among children in rural Orissa, India in villages where the Government of India's Total Sanitation Campaign had been implemented at least three years prior to the study.

**Methods and Findings:**

We conducted surveys with heads of 136 households with 145 children under 5 years of age in 20 villages. We describe defecation and feces disposal practices and explore associations between safe disposal and risk factors. Respondents reported that children commonly defecated on the ground, either inside the household (57.5%) for pre-ambulatory children or around the compound (55.2%) for ambulatory children. Twenty percent of pre-ambulatory children used potties and nappies; the same percentage of ambulatory children defecated in a latrine. While 78.6% of study children came from 106 households with a latrine, less than a quarter (22.8%) reported using them for disposal of child feces. Most child feces were deposited with other household waste, both for pre-ambulatory (67.5%) and ambulatory (58.1%) children. After restricting the analysis to households owning a latrine, the use of a nappy or potty was associated with safe disposal of feces (OR 6.72, 95%CI 1.02–44.38) though due to small sample size the regression could not adjust for confounders.

**Conclusions:**

In the area surveyed, the Total Sanitation Campaign has not led to high levels of safe disposal of child feces. Further research is needed to identify the actual scope of this potential gap in programming, the health risk presented and interventions to minimize any adverse effect.

## Introduction

Millennium Development Goal (MDG) target 7c includes the reduction by half of the proportion of the population without sustainable access to basic sanitation by 2015 [1]. This MDG is far off track from being met; indeed 2.5 billion people were still without access to improved sanitation by the end of 2011 [2]. In India, sanitation represents a particular challenge, as 50% of the population still practice open defecation (which, by definition, includes disposals with solid waste) and only 35% of the population uses improved sanitation [2].

This gap in access to improved sanitation has led to large-scale interventions to increase sanitation coverage, in some cases without a corresponding focus on use. The largest rural sanitation campaign is the Nirmal Bharat Abhiyan in India, previously known as the Total Sanitation Campaign (TSC), a subsidy-based approach that seeks to create demand and provide subsidies to below the poverty line (BPL) households towards construction of individual household latrines [3]. The TSC reported building one latrine per 10 rural people in India between 2001 and 2011, and there is some evidence that this has resulted in health gains [4]. There is also evidence, however, that actual use of the latrines is suboptimal, and in many cases is isolated to the adult female members of the household [5]–[8]. Yet both coverage and use of sanitation are necessary to reduce the exposure to feces in the environment and yield reductions in enteric diseases [9].

Another aspect of suboptimal sanitation is the improper collection and disposal of child feces. While there are few published studies, the evidence suggests that in many low-income settings, nappies (i.e. diapers or cloth) and potties are rarely available or used, making the hygienic collection of young children's feces difficult; if collected, such feces are often disposed of in a manner that does not prevent further exposure to household members or contamination of water sources [10].

In fact, the unsanitary disposal of child feces may present a greater health risk than that of adults. First, young children represent the highest incidence of enteric infections [11], and their feces are most likely to contain agents [12]. Second, young children tend to defecate in areas where susceptible children could be exposed [13]. Third, young children who are also most at risk of mortality and the serious sequelae associated with enteric infection [14], [15] are most likely to be exposed to these ambient agents due to the time they spend on the ground, their tendency to put fingers and fomites in their mouths, and common behaviors such as geophagia [16], [17]. In a meta-analysis of 10 observational studies published between 1987 and 2001, Gil et al. (2004) found that child feces disposal behaviors considered risky (open defecation, stool disposal in the open, stools not removed from soil, stools seen in household soil, and children seen eating feces) were associated with a 23% increase in risk of diarrheal diseases (RR 1.23, 95%CI 1.15–1.32); behaviors considered safe (use of latrines, nappies, potties, toilets, washing diapers) were borderline protective (RR 0.93, 95%CI 0.86–1.00) [10]. In addition, improved disposal of child feces could have an impact on enteric infections other than diarrhea; a study in rural Bangladesh found that the disposal of child feces in closed spaces such as pit latrines resulted in a 35% reduction in helminthiasis in children under 2 compared with disposal in open space [18].

In connection with a large scale trial to assess the effectiveness of rural sanitation in Orissa State [9], we undertook this study to describe the practices with respect to the disposal of feces of children under 5 years old in rural villages where the TSC had been implemented at least 3 years prior to this study.

## Methods

### Study design and setting

The study followed a cross-sectional design. It was conducted in June and July 2012 in Puri District, a coastal region of the State of Orissa in Eastern India. A sample of 20 villages was selected randomly from a list of 35 villages where the TSC had been implemented by a partner NGO of WaterAid India (the implementer of the large scale trial) at least 3 years prior to the study. This study was a component of a larger study on latrine coverage and use by adults which contains further details on the study setting [7].

### Household selection

In the selected villages, all households were eligible for inclusion in the study. For logistical reasons, we targeted 20 households in each of the 20 villages that were selected in a larger study assessing latrine coverage and use [7]. The sample size was chosen for logistical reasons without conducting power calculations. Households eligible for inclusion in this study were required to have at least one child under five years old, which led to a sample of 136 households out of the 447 households that were surveyed in the larger study [7]. Households were selected using systematic sampling following the method described by the Extended Program on Immunization (EPI) [19]. This approach consists of spinning a pen in a central location of the village to determine the direction in which the enumerator would sample households. Each of three enumerators enrolled every other household in that direction until they reached their quota of 7 households or the village boundary was reached. In the case when the village boundary was reached before the quota was met, the enumerator would start the process again from the central location. The actual number of households enrolled varied slightly among villages due to logistical constraints. Households were enrolled only after receiving all the details concerning the study and consenting to participate. Respondents were female heads of household or, if unavailable, male heads of households or an adult over 18 years of age. Households where no adults were present at the time of visit or that did not consent to participate in the study were not enrolled.

### Survey tool

Data collection tools included a structured survey and spot-checks of household latrines looking for indicators of use and of the compound looking for the presence of human stools. The survey was developed in English, translated to Oriya (the local language) and then back-translated to assess accuracy. Fluent Oriya speakers conducted the survey, which included questions on demographics, type of household construction, education level of heads of households, ownership of a latrine and distance to nearest water source to use in the latrine. The outcomes of interest were defecation sites of children under 5 and feces disposal sites. We assessed child feces disposal practices based on the wording used in the core questions of the WHO/UNICEF Joint Monitoring Programme on Water and Sanitation (JMP) [20]: “The last time this child [youngest child in mobility category] passed stools, what was done to dispose of the stools?” The questions on defecation and disposal practices were asked for the youngest child in each household in each of the two mobility categories: pre-ambulatory children (worded as “child that cannot yet walk” in the questionnaire) and ambulatory children (worded as “child that can walk”). As such, data from a total of two children per household were possible.

### Data analysis

Data were entered using EpiData 3.1 (EpiData Association, Odense, Denmark) and analyzed using STATA version 12 (StataCorp, College Station, Texas, United States). For univariate descriptive statistics, analysis was stratified by mobility category. Feces disposal was recoded into a binary outcome, “safe” and “unsafe,” based on whether the reported behavior was expected to be associated with the fecal contamination of the environment [21]. We used the JMP definition of safe disposal (defecation into a latrine, disposal of stools in a latrine or buried) to categorize behaviors as “safe” [20]. Seven values were missing for disposal site when the site of defecation of the child was an open field or roadside; these unknowns were categorized into the unsafe disposal category.

Bivariate analysis between safe feces disposal and defecation site, household characteristics and latrine ownership were conducted using logistic regression. Since not owning a latrine predicts failure to safely dispose feces (only those households with a latrine reported safe disposal of child feces), we restricted subsequent regression analyses quantifying the relationship between potential determinants and safe disposal of child feces to households owning a latrine. In order to adjust for clustering of children within households, we used generalized estimating equations with robust standard errors. Due to the small sample size, it was not possible to conduct multivariate analysis to adjust for potential confounders.

### Ethics Statement

This study was approved by the Ethics Committees of the London School of Hygiene and Tropical Medicine (United Kingdom) and Xavier Institute of Management, Bhubaneswar (India), who also approved the consent procedures. Prior to enrollment, field workers fluent in Oriya read an information sheet describing the study, answered any questions and asked for written consent to participate, The study participants received no compensation for their participation. Anonymity was ensured through the use of household identification numbers and no names were recorded.

## Results

Although a total of 447 households were enrolled into the larger study [7], only 136 households reported to have a child below the age of five and thus met the eligibility criteria to participate in this sub study. A total of 145 children from 136 households are reported on in this study, of these forty (27.6%) were pre-ambulatory. Thirty-three (82.5%) pre-ambulatory children and 81 (77.1%) ambulatory children came from a household with a latrine (table 1).

**Table 1:** Household characteristics of participating pre-ambulatory and ambulatory children.

Characteristics	Pre-ambulatory (n = 40)		Ambulatory (n = 105)	
	N	%	N	%
**Ownership of a latrine**				
Yes	33	83	81	77
No	7	18	24	23
**Water access to use in latrine** 1				
Water on premise	28	70	67	64
Water not on premise	5	13	13	12
**Number of persons per household**				
1–3	0	0	3	3
4–6	18	46	50	48
7–9	9	23	33	31
10+	12	31	19	18
**Religion**				
Hindu	40	100	101	97
Muslim	0	0	3	3
**Education of male head of household**				
Illiterate	3	8	9	9
Literate no formal schooling	2	5	13	13
Some or completed primary school	7	18	23	22
Some or completed secondary school	25	63	45	43
Any level of higher education	3	8	9	9
**Education of female head of household**				
Illiterate	8	20	27	26
Literate no formal schooling	6	15	13	13
Some or completed primary school	10	25	25	24
Some or completed secondary school	12	30	32	31
Any level of higher education	4	10	5	5
**Type of house construction** 2				
Pucca	27	68	57	54
Semi-Pucca	10	25	29	28
Kuchha	3	8	19	18
**Own a BPL card**				
Yes^3^	30	81	62	65
No	7	19	34	35

1 only among households with latrines.

2 Pucca  = concrete; Kuccha  = mud and dung.

3 checked or reported.

The defecation and disposal sites reported for the last time the children defecated are listed in tables 2 and 3. Most children were reported to defecate on the ground, either inside the home (57.5%) or compound (20.0%) for pre-ambulatory children, or inside the compound for ambulatory children (55.2%). Twenty percent of pre-ambulatory children used potties (17.5%) and nappies (2.5%), while 20.0% of ambulatory children defecated in a latrine. The defecation sites of children were categorized as improved if the child defecated in a potty or nappy or unimproved if they defecated on paper, roadside, inside compound, inside household or in an open field.

**Table 2:** Frequency of feces disposal sites of pre-ambulatory children by site of defecation (n = 40).

	Defecation sites					
	Potty	Nappy	On paper	Ground in compound	Ground inside household	Total
**Disposal sites**	7 (18)	1 (3)	1 (3)	8 (20)	23 (58)	40 (100)
Latrine	1 (14)	0 (0)	0 (0)	1 (13)	2 (9)	4 (10)
Garbage	6 (86)	1 (100)	0 (0)	6 (75)	14 (61)	27 (68)
Field	0 (0)	0 (0)	0 (0)	0 (0)	1 (4)	1 (3)
Left in the open	0 (0)	0 (0)	1 (100)	1 (13)	2 (9)	4 (10)
Washed*	0 (0)	0 (0)	0 (0)	0 (0)	3 (13)	3 (8)
Roadside	0 (0)	0 (0)	0 (0)	0 (0)	1 (4)	1 (3)

*Includes: washing, washing clothes, and cleaning it in water.

**Table 3:** Frequency of feces disposal sites of ambulatory children by site of defecation (n = 105).

	Defecation sites							
	Latrine	Potty	On paper	Roadside	Ground in compound	Ground inside household	Open field	Total
**Disposal sites**	21 (20)	1 (1)	4 (4)	9 (9)	58 (55)	5 (5)	7 (7)	105 (100)
Latrine	21 (100)	1 (100)	0 (0)	0 (0)	0 (0)	0 (0)	0 (0)	22 (21)
Garbage	0 (0)	0 (0)	2 (50)	6 (67)	49 (84)	4 (80)	0 (0)	61 (58)
Field	0 (0)	0 (0)	0 (0)	1 (11)	1 (2)	0 (0)	1 (14)	3 (3)
Buried	0 (0)	0 (0)	1 (25)	0 (0)	0 (0)	0 (0)	0 (0)	1 (1)
Left in the open	0 (0)	0 (0)	1 (25)	1 (11)	8 (14)	1 (20)	0 (0)	11 (10)
Unknown	0 (0)	0 (0)	0 (0)	1 (11)	0 (0)	0 (0)	6 (86)	7 (7)

The feces of most children ended up in the household's solid waste disposal site typically located outside at the rear of the compound (“garbage”), both for pre-ambulatory (67.5%) and ambulatory (58.1%) children. Overall, the feces of only 10.0% of pre-ambulatory children and 21.9% of ambulatory children were reported to have been safely disposed of, which was defined as either directly defecating in a latrine or feces being rinsed/put in a latrine or buried [20]. Although 84 (80.0%) defecation events of ambulatory children occurred outside of the latrine, the feces were only disposed of in a latrine once (1.2%) and buried once (1.2%).

Safe disposal of child feces only occurred in households that owned latrines (n = 106). As such, it was not possible to conduct analysis on determinants of safe disposal in non-latrine households. However, latrine ownership was no guarantee of safe disposal of child feces: the feces of only 27 (23.7%) children from 26 (24.5%) households with latrines were reported to be safely disposed of. In households with latrines that reported safely disposing of their children's feces, no human stools were observed in the compound during spot check observations. In households with latrines that reported safely disposing of their children's feces, 19 (73.1%) had wet floors in the latrine and 18 (69.2%) had cleaning products in their latrines, both of which are positive indicators of latrine use.

In the crude bivariate analysis (data not presented in tables) one variable was found to be associated with safe child feces disposal: defecation in a potty or nappy (Odds Ratio [OR] 7.91, 95% confidence interval [CI] 1.24–50.41). This may be linked to household education level, household wealth/socioeconomic status, and/or local availability of potties or nappies, but these could not be controlled for in multivariate analysis due to small sample size. After restricting the analysis to households owning a latrine, defecation by children into a potty or nappy remained associated with safe stool disposal (Table 4). While safe disposal of child feces was higher when children used potties or nappies (OR 6.72, 95%CI 1.02–44.38), the feces of the majority (75%) of children defecating in potties or nappies were still not safely disposed and the observed association could be due to confounders which could not be adjusted for in the analysis.

**Table 4:** Bivariate analysis assessing association between household characteristics and safe disposal of child feces among households with a latrine (n = 114 children from 106 households).

	N	Total	%	OR	95% CI	P-value^1^
**Mobility Category**						
Pre-ambulatory	4	33	12	Ref.	-	-
Ambulatory	23	81	28	3.21	1.00–10.31	0.05
**Defecation site**						
Unimproved^2^	4	85	5	Ref.	-	-
Improved^3^	2	8	25	6.72	1.02–44.38	0.05
**When the latrine was built**						
<3 years ago	3	23	13	Ref.	-	-
3–5 years ago	3	31	10	0.74	0.13–4.09	0.73
>5 years	21	58	36	3.77	0.99–14.33	0.05
**Water access to use in latrine**						
Water not on premise	1	18	6	Ref.	-	-
Water on premise	26	94	28	6.16	0.76–49.72	0.09
**Number of persons per household**						
10+	6	25	24	Ref.	-	-
7–9	10	33	30	1.40	0.43–4.61	0.58
4–6	10	53	19	0.77	0.25–2.36	0.65
1–3	1	2	50	3.25	0.18–60.29	0.43
**Religion**						
Hindu	25	111	23	Ref.	-	-
Muslim	2	3	67	6.92	0.59–80.56	0.12
**Education of male head of household** 4						
Illiterate 5	0	8	0	-	-	-
Literate no formal schooling	2	10	20	Ref.	-	-
Some or completed primary school	4	24	17	0.80	0.12–5.21	0.82
Some or completed secondary school	15	56	27	1.46	0.27–7.85	0.66
Any level of higher education	5	11	45	3.33	0.47–23.72	0.23
**Education of female head of household**						
Illiterate	5	25	20	Ref.	-	-
Literate no formal schooling	2	12	17	0.81	0.13–4.91	0.82
Some or completed primary school	9	26	35	2.12	0.58–7.73	0.25
Some or completed secondary school	8	41	20	1.00	0.29–3.47	1.00
Any level of higher education	3	9	33	2.02	0.37–10.99	0.42
**Type of house construction**						
Pucca	19	70	27	Ref.	-	-
Semi-Pucca	4	31	13	0.40	0.12–1.33	0.13
Kuchha	4	13	31	1.15	0.33–4.03	0.83
**Own a BPL card**						
Yes^6^	17	79	22	Ref.	-	-
No	9	27	33	1.95	0.73–5.22	0.18

1 Wald test.

2 Paper, roadside, inside compound, inside household, in open field.

3 Potty, nappy.

4 used robust standard errors without GEE as not possible.

5 dropped from analysis.

6 checked or reported.

Note 1: Denominators vary as not all respondents answered all questions.

Note 2: Due to the small sample size of the study and the rare occurrence of safe feces disposal, it was not possible to conduct multivariate analysis beyond restricting the analysis to households owning a latrine, therefore these crude odds ratios should be interpreted cautiously.

Safe stool disposal was weakly associated with ambulatory mobility category, owning a latrine for more than 5 years compared to less than 3 years and water on premise to use in latrine. The safe disposal of child feces was higher in ambulatory children than in pre-ambulatory children after restricting the analysis to households owning a latrine (OR 3.21, 95%CI 1.00–10.31) due to ambulatory children defecating directly into a latrine. The feces of ambulatory children that defecated outside of the latrine were only safely disposed of twice (2.4%) compared to four (10.0%) pre-ambulatory children's feces being disposed of safely. Households that had a latrine for more than five years were more likely to dispose of their child's feces safely than households that built their latrines less than three years ago (OR 3.77, 95%CI 0.99–14.33). Having owned a latrine for between 3 and 5 years was not associated with safer stool disposal (OR 0.74, 95%CI 0.13–4.09). Most of the children whose feces were reported to being safely disposed came from households (96.0%) with water on the premises. Water on the premises increased the odds of safe disposal (OR 6.16, 95%CI 0.76–49.72), although not significantly.

## Discussion

We describe reported defecation and disposal practices of 145 children under five years old from 136 households in rural Orissa, together with factors associated with these practices. We found that most child feces are disposed of unsafely even among households with latrines.

Most child feces ended up in the household waste disposal site. Such disposal is considered “open defecation” under the definitions used by the JMP [2]. In these communities, household waste is generally collected in piles or pits and mostly located in the backyard of the house and according to qualitative research it is sometimes burned. This practice could create a source of pathogen exposure, either directly through leaching or dispersion with the rains or indirectly via animals and mechanical vectors (flies), and its proximity to households may increase the risk compared to the more typically distant open defecation sites. However, the actual risk that this practice presents has not been quantified.

In this study population, safe disposal of child feces was limited almost exclusively to latrine use by ambulatory children. Few caregivers collected and disposed of stools around the compound safely. As data was not collected on the age of the children within the mobility categories, it is not possible to know whether there was an association between age and latrine use, which may explain the ambulatory children that did not use the latrine for defecation. Defecation in potties or nappies, though uncommon, was associated with safe disposal of the feces even though the majority of the feces collected in potties or nappies were still disposed of unsafely. Studies in Burkina Faso and Peru where defecation in a potty was more common in the study population also found that defecation into a potty was associated with safe disposal of the stools into a latrine [21], [22].

Longer-term adoption of a latrine by households (>5 years) was weakly associated with safer stool disposal. It is possible that these households built their latrines themselves as it was in the early stages of the TSC and so they may attach more priority to sanitation generally, it seems likely that household investment in sanitation would increase use of the latrine. Alternatively, households may take more time to adopt safe child feces disposal practices after they own their latrines, though the possible association could be due to other confounders not explored or adjusted for in this paper such as wealth, exposure to sanitation messages and use of the latrine by other members of the family.

Access to water within the compound was found to be associated with safe child feces disposal in Burkina Faso [21]. While our findings were suggestive of an association, our sample size may have been too small to achieve statistical significance. Curtis and colleagues hypothesized that this association was maybe due to mothers in households with improved water sources wanting to conform to better standards of hygiene behavior or due to increased time to carry out safer behaviors [21].

The study involved a small sample from a single, non-randomly selected district in Orissa State, and thus cannot be generalized beyond the study population itself. Nevertheless, our findings are similar to those from large-scale surveys in India. The latest Demographic and Health Survey (DHS) for India (2005–2006) reported that nationally, 79.0% percent of child feces were disposed of unsafely [23] compared to our finding of 81.4%. In that DHS survey, Orissa was found to have one of the lowest percentages in the country of safe child stool disposal, with only 7.0% of the stools being disposed of safely [23]. The main disposal methods in Orissa were found to be leaving the feces in the open (53.7%) or disposing of them in the garbage (32.3%). These methods were also among the ones found to be most common in our study. A more recent but smaller study conducted in 6 states in India (not including Orissa), reported 55.0% safe stool disposal practices [5].

India may present a particular challenge for the safe disposal of child feces owing to the continuing widespread practice of open defecation in the country [2]. However, our results are largely consistent with previous research in other countries, particularly in Asia [10]. Studies analyzed by Gil and colleagues (2004) found low use of direct defecation into latrines and of potties and diapers as defecation sites in Asia. The review authors also reported that the disposal of child feces in latrines was uncommon in studies from Asia (three studies with a prevalence of <25%). In Africa or Latin America, the behavior is more widespread with a prevalence of child feces disposal in latrines of more than 50% [10].

Although we present data on pre-ambulatory and ambulatory children, there were notably fewer data on pre-ambulatory children than ambulatory children, as the latter category encompasses more possible ages under five. This limits the conclusions that can be inferred from this data about the different mobility categories. In future studies, the sampling procedure should take this into account as well as record the actual ages of the children. Moreover, in accordance with practices in this setting, we targeted the survey to the female head of household but accepted responses from the male head if she was not available. Future surveys may wish to explore targeting the child's principal caregiver.

Like the DHS survey, we relied on reported practices via a survey rather than direct observation, although surveys are susceptible to courtesy and recall bias [24], [25]. Gil and colleagues found greater precision among studies employing spot checks and structured observations rather than questionnaires [10] so our study survey results should be interpreted with some caution. However, direct observation of sanitation practices has been shown to be subject to reactivity (Hawthorne effect) in the study population [9]. Like the DHS survey, we endeavored to minimize reporting bias by enquiring about the “last time” rather than a usual practice for disposal of child feces [24]. While we cannot rule out courtesy bias, adjustment for an exaggeration of positive (safe) behaviors would further reduce the already low level of safe feces disposal that we report here. Due to the small sample size of the study and the rare occurrence of safe feces disposal, it was not possible to conduct multivariate analysis beyond restricting the analysis to households owning a latrine, which is an important determinant of safe feces disposal [26]–[29]. The associations that were found in the bivariate analysis should thus be interpreted cautiously as they are likely to be confounded by other variables.

Despite these limitations, this study draws attention to unsafe disposal of child feces in this area of India and adds to a growing body of evidence raising questions about the effectiveness of sanitation strategies focused on expanding coverage without a corresponding emphasis on optimizing use. The larger study in the same households as those investigated here, reported low levels of latrine use by many adults [7]. These and other studies reporting on deficiencies in latrine use in India [5], [8] suggest that current sanitation campaigns in rural India may be more effective in addressing coverage than securing the behavior change necessary to ensure the safe disposal of feces of all members of the household in a manner that minimizes exposure to human feces—a condition to optimizing health gains.
